# PLEKHA4 Is a Prognostic Biomarker and Correlated with Immune Infiltrates in Glioma

**DOI:** 10.1155/2023/4504474

**Published:** 2023-01-17

**Authors:** Wei Zhang, Liu Li, Piao-Piao Bian, Qiu-Ping Luo, Zhong-Tang Xiong

**Affiliations:** ^1^Department of Pathology, Third Affiliated Hospital, Guangzhou Medical University, Guangzhou 510150, China; ^2^Department of Gastroenterology, Third Affiliated Hospital, Guangzhou Medical University, Guangzhou 510150, China; ^3^Key Laboratory of Major Obstetric Diseases of Guangdong Province, The Third Affiliated Hospital, Guangzhou Medical University, Guangzhou 510150, China

## Abstract

**Objective:**

Gliomas are the most common and life-threatening intracranial tumors. Immune infiltration of the tumor microenvironment significantly affects tumor prognosis in glioma. Recently, PLEKHA4 was reported to be upregulated in melanoma and closely associated with tumor genesis and development, but its role in glioma is poorly understood. Our aim was to investigate the expression, functional role, and prognostic value of PLEKHA4 in glioma.

**Methods:**

The expression levels of PLEKHA4 in 33 types of cancer in the TCGA (The Cancer Genome Atlas) database were collected via the UCSC Xena browser. The clinical samples of glioma patients were downloaded from the TCGA database. Immunohistochemistry was used to verify PLEKHA4 expression in tumor tissues. We assessed the influence of PLEKHA4 on survival of glioma patients by survival module and GEPIA. Then, we downloaded datasets of glioma from TCGA and investigated the correlations between the clinical characteristics and PLEKHA4 expression using logistic regression. Moreover, we used TIMER to explore the collection of PLEKHA4 expression and immune infiltration level in glioma and to analyze cumulative survival in glioma. Gene Set Enrichment Analysis (GSEA) was performed using the TCGA dataset.

**Results:**

PLEKHA4 transcript levels were significantly upregulated in multiple cancer types, including gliomas. Moreover, immunohistochemical analysis verified that PLEKHA4 was overexpressed in gliomas compare to the corresponding normal tissues. Univariable survival and multivariate cox analysis show that increased PLEKHA4 expression significantly correlated with age, tumor grade, IDH mutation status, and 1p/19q codel status, and higher PLEKHA4 had shorter OS, DSS, and PFI. Specifically, PLEKHA4 expression level had significant positive correlations with infiltrating levels of B cell, CD4+ T cells, CD8+ T cells, macrophages, neutrophils, and DCs in glioma, and upregulation of PLEKHA4 expression was significantly related to immune cell biomarkers and immune checkpoint expression in glioma. In addition, several GO and Kyoto Encyclopedia of Genes and Genomes (KEGG) items associated with immune response, JAK STAT signal pathway, and cell cycle were significantly enriched in the high PLEKHA4 expression phenotype pathway.

**Conclusions:**

Our findings proposed that PLEKHA4 was an independent prognostic biomarker and correlated with immune infiltrates in glioma, and targeting PLEKHA4 might improve immunotherapy in glioma. Of course, these findings also need basic experiments and further clinical trials to confirm in the future.

## 1. Introduction

Glioma is the most common aggressive and lethal tumor in central nervous system and is the predominant brain primary malignancy [1]. According to the World Health Organization, gliomas are classified into histological grades I-IV, and glioblastoma (GBM), the WHO IV grade glioma, has the worst prognosis [2]. Tumor grading and biological behavior of glioma influence the patient's therapeutic schedules, management plans, and the prognosis [3, 4]. Presently, the surgery with radiotherapy and chemotherapy is the mainstream treatment of glioma [5]. Based on traditional histological classification, molecular parameters are closely related to the diagnosis, treatment, and prognosis of glioma, including 1p/19q codeletion status; isocitrate dehydrogenase 1(IDH1) mutation, H3G34 mutation, and H3K27 mutation are included in the 2021 World Health Organization Classification of Tumors of the Central Nervous System [6]. The discovery of these molecular markers can help us better understand the underlying molecular mechanism of glioma, thus beneficial to better clinically diagnose and treat gliomas [7]. Molecular markers show that the prognosis of glioma is complex, and a single index cannot accurately predict tumor prognosis. The combined analysis of multiple indicators will improve the accuracy of prognosis prediction [7–9]. The survival status of different glioma patients is also different, so the pursuit of individualized treatment is one of the clinical goals [10].

In recent years, the relationship between tumor immune microenvironment and immunotherapy has received more and more attention [11, 12]. Immune checkpoint blockers, such as CTLA-4 and PD-1/PD-L1 inhibitors, promote the development of tumor immune response to glioma, enhancing the unique role of the tumor immune system response [13–15]. Tumor microenvironment (TME) plays an important role for tumor development and progression and depends on the mutual effect among the cancer cells, immune system, and tumor microenvironment [16–18].Tumor-infiltrating immune cells (TIIC) can affect prognosis and therapeutic efficiency of patients [19, 20]. There are various immune and nonimmune components in the tumor microenvironment and their consequences of the efficacy of immunotherapies [18]. Hence, it is essential to evaluate the immunological characteristics of TME and characterize glioma to identify novel biomarkers for predictions and molecules related to immunity.

PLEKHA4, phosphoinositide binding protein PLEKHA4 (pleckstrin homology, including family A, number 4), mediates the activity of the CUL3–KLHL12 E3 ligase of polyubiquitinated DVL [21, 22]. PLEKHA4 acts as a role of sequestering the substrate-specific linker KLHL12 in the plasma membrane related clusters, thereby reducing DVL ubiquitination, increasing DVL levels, and activating Wnt/*β*-catenin signaling in mammalian cells. It has been reported that PLEKHA4 is highly expressed in melanoma [23, 24]. Upregulated PLEKHA4 promotes Wnt/*β*-catenin signaling-mediated G1/S transition and proliferation in melanoma [25]. However, the prognostic significance of PLEKHA4 and its correlation with immune infiltrates in glioma remain unclear. According to data analysis, a lot of potential tumor markers can be unearthed for the study of antitumor therapy. In the present study, we explored the expression levels of PLEKHA4 in 20 types of human cancers and corresponding normal tissue and unearthed the prognostic value of PLEKHA4 in glioma based on data from The Cancer Genome Atlas (TCGA). The results of this study indicated that PLEKHA4 expression was a prognostic factor and related to the clinicopathological characteristics of glioma patients. Furthermore, we determined the potential relationship between PLEKHA4 and immune cell infiltration, immune cell biomarkers, or immune checkpoints in the glioma microenvironment through the tumor immunity estimation resource (TIMER). Eventually, Gene Set Enrichment Analysis (GSEA) was carried out to evaluate gene sets enriched in the PLEKHA4 high and PLEKHA4 low expression groups and the biological processes associated with PLEKHA4. Taken together, our findings indicate that upregulation of PLEKHA4 expression is associated with poor prognosis and tumor immune infiltration in glioma.

## 2. Materials and Methods

### 2.1. Data Collection and Bioinformatics Analysis

Collection of PLEKHA4 data is from the TCGA (https://tcga-data.nci.nih.gov/tcga/) and the UCSC Xena (https://xenabrowser.net/datapages/) by the Toil process [26], unified handling TCGA, GTEx, and TPM RNA-seq data format. The gliomas of TCGA and the corresponding normal tissue data of GTEx were extracted. The expression profile of PLEKHA4 was extracted from TCGA RNA-seq data of 703 glioma patients [27]. By removal control/normal (not all items have control/normal) and reservation of clinical information, the corresponding clinical prognosis information (overall survival (OS), disease-specific survival (DSS), and progress free interval (PFI)) was obtained from above dataset. Glioma patient datasets, including gene expression profiles and clinical information, were downloaded from the publicly available TCGA [28]. Then, RNA-sequencing data were firstly transformed to convert count data to value more similar to those resulting from microarrays. We converted the data related to the PLEKHA4 from the high-throughput sequencing fragments per kilobase per million (HTSeq-TPKM) format to the transcripts per kilobase million format with the preservation. In this study, clinical data and prognostic information of all available samples were extracted, and prognostic indicators mainly included OS, DSS, and PFI. Forty formalin-fixed paraffin-embedded tissues were obtained from the archives of Department of Pathology, Third Affiliated Hospital, Guangzhou Medical University (Guangzhou, China); the choice of glioma samples was based on the several factors: the availability of resected tissue, no radiotherapy, and chemotherapy before surgery. The clinical specimens were used for this study with the consent of the patients or their relatives according to the hospital's ethics committee. The study protocol was approved by the Ethics Committee of the Third Affiliated Hospital of Guangzhou Medical University.

### 2.2. Oncomine

The Oncomine (http://www.oncomine.org) is a gene expression array dataset and a public, available, online cancer microarray database convenient to research from genome-wide expression analyses [29]. The Oncomine is performed to evaluate the mRNA level of PLEKHA4 during different cancers vs normal tissues and in various types of gliomas. The thresholds were limited as *P* value ≤ 1E-2, gene rank top 1%, and fold − change ≥ 2 − fold.

### 2.3. Survival and Expression Analysis by GEPIA

Gene Expression Profiling Interactive Analysis (http://gepia.cancer-pku.cn/index.html) (GEPIA) is an online analysis tool, which can analyze 9,736 tumors from the RNA sequencing expression data and 8,587 normal samples from projects known as the GTEx, and TCGA deals with a standard method [30]. Survival curves of differential PLEKHA4 expression were performed to explore the correlation of the gene expression with glioma patients' prognosis by GEPIA. In addition, the GEPIA database was used to detect the correlation between PLEKHA4 in glioma and immune cell biomarkers or immune checkpoints.

### 2.4. Human Protein Atlas (HPA)

Human Protein Atlas (HPA) (https://www.proteinatlas.org) is an accessible online database which contains maps of all known human proteins in cells, tissues, and organs by integration of data from various omics technologies, including antibody-based imaging, transcriptomics, systems biology, and mass-spectrometry-based proteomics [31, 32]. In this study, we used HPA database to determine PLEKHA4 protein expression in gliomas and normal brain tissues.

### 2.5. Analysis of Gene Set Enrichment

GSEA (http://software.broadinstitute.org/gsea/index.jsp) is a computational method verifying whether a priori presetting of genes indicates statistically significant, concordant differences between two biological states [33]. In order to explore the biological pathways involved in glioma progression, 703 glioma samples for GSEA were downloaded from TCGA database. These samples were divided into a PLEKHA4 high expression group (*n* = 351) and a PLEKHA4 low expression group (*n* = 352) based on the median of PLEKHA4 expression as a cut-off point, and the number of permutations was 1000. Expression profiles of PLEKHA4 were used as phenotypic labels, and we used nominal *P* values and normalized enrichment scores (NES) to rank the pathways with PLEKHA4 enrichment in each phenotype.

### 2.6. Immune Cell Infiltration Analysis via TIMER

TIMER (https://cistrome.shinyapps.io/timer/) applies a previously published statistical method called deconvolution, which uses gene expression profiles to infer the number of tumor infiltrating immune cells (TIIC) [34]. TIMER database contains 10,897 samples across 32 cancer types provided by The Cancer Genome Atlas, which are applied for the approximation of immune infiltrates. A series of analysis on the expression of PLEKHA4 in different types of cancer and its correlation with the abundance of immune infiltrates was performed. These immune infiltrates include B cells, CD4+ T cells, CD8+ T cells, neutrophils, macrophages, and dendritic cells through gene modules [35]. In addition, TIMER is used to analyze the correlation between the expression level of PLEKHA4 in glioma and the expression level of immune checkpoints.

### 2.7. H&E and Immunohistochemical Staining

The glioma paraffin specimens were cut and subjected to H&E staining using routine procedures. Immunohistochemistry (IHC) was performed on formalin fixed, paraffin-embedded tissue sections using a two-step protocol. The PLEKHA4 was detected using the rabbit anti-PLEKHA4 polyclonal antibody NBP2-47331 (Novus Biologicals, Inc., United States). Briefly, pressure cooker-mediated antigen retrieval was performed in EDTA buffer pH 9.0 for 8 minutes. Sections were incubated with a 1 : 150 dilution of anti-PLEKHA4 antibody at 37°C for 2 hours. After 2 hours, the sections were washed and incubated with the secondary antibody for 30 minutes at room temperature. Finally, the slides were developed using a DAB chromogen kit and counterstained with Mayer's hematoxylin [36].

### 2.8. Statistical Analysis

The statistical data obtained from TCGA is consolidated and implemented by R-3.6.3, using logistic regression to analyze the correlation between clinical information and PLEKHA4 expression. Survival curves were constructed using the Kaplan-Meier method, and the differences between the survival curves were examined by the log-rank test. Univariate Cox proportional hazard regressions were applied to estimate the individual hazard ratio (HR) for DSS, PFI, and OS. The significant variables in the univariate analyses (*P* < 0.05) were then put into the multivariate analysis. The HR with 95% confidence interval (CI) was measured to estimate the hazard risk of individual factors. All reported *P* values were two sided, and *P* < 0.05 was considered statistically significant.

## 3. Results

### 3.1. The mRNA and Protein Expression Levels of PLEKHA4 in Glioma

**Figure 1:**
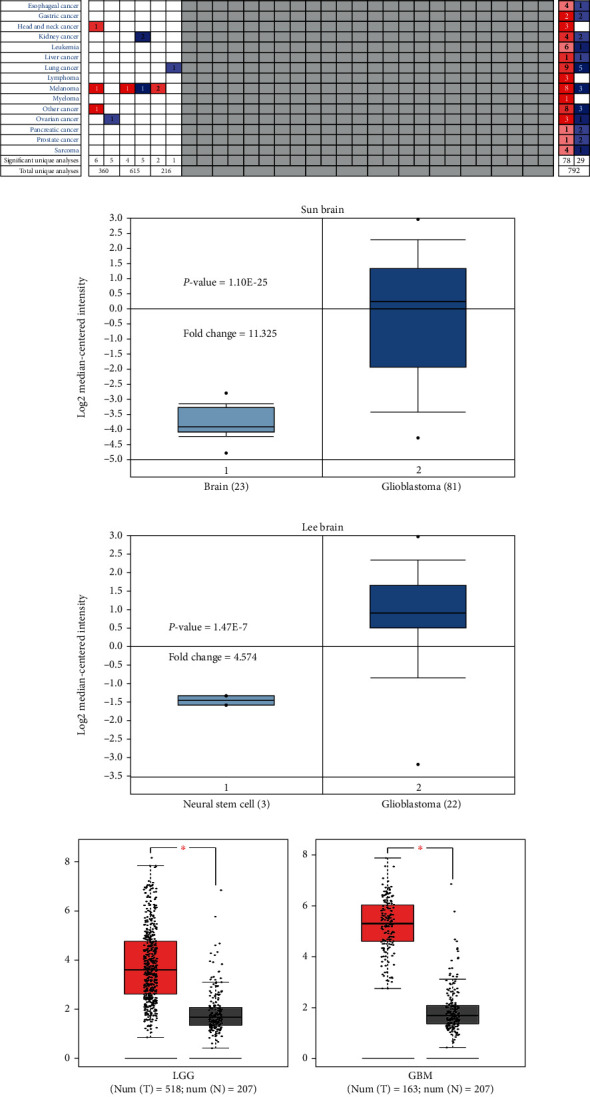
PLEKHA4 is overexpressed in several cancers including glioma. (a) Summary of PLEKHA4 expression analyses in multiple cancer types and their corresponding normal tissues. Red color indicates high PLEKHA4 expression in the corresponding cancer and blue color indicates low PLEKHA4 expression in the corresponding cancer. (b) PLEKHA4 expression in Sun brain (normal brain vs. glioblastoma) dataset was shown. (c) PLEKHA4 expression in Lee brain (neural stem cell vs. glioblastoma) dataset was shown. Note: *P* < 0.01 indicates statistical significance; PLEKHA4 was among the top 1% overexpressed genes in glioma. (d, e) PLEKHA4 transcript expression levels in low-grade glioma (LGG; red; *n* = 518), glioblastoma multiforme (GBM; red; *n* = 163), and corresponding normal brain tissues (black; *n* = 207) from the GEPIA datasets.

**Figure 2:**
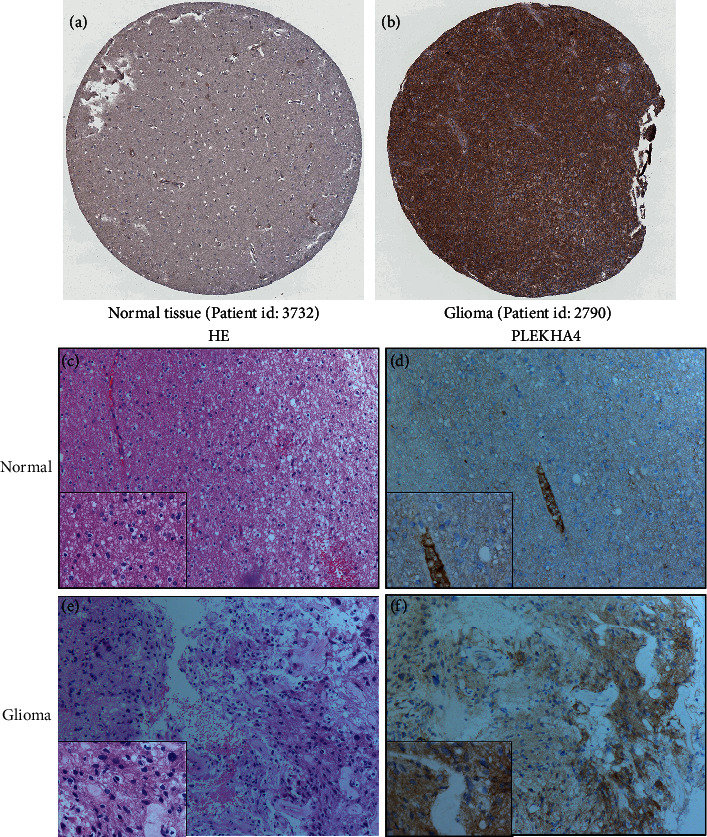
Immunohistochemical staining. (a, b) Representative IHC-stained brain section images from the HPA database show PLEKHA4 expression in normal healthy individual (patient id: 3732) and glioma patient (patient id: 2790). Brown staining represents anti-PLEKHA4 antibody staining. (c, d) H&E-stained slides and IHC showed expression of PLEKHA4 in their normal controls. (e, f) H&E-stained slides and IHC showed expression of PLEKHA4 in glioma tissues. Original magnifications ×100 and ×400 (lower panels), EnVision Method.

At first, we compared the mRNA expression of PLEKHA4 in 20 types of cancer with that in normal tissues through Oncomine databases. The analysis showed that the expression of PLEKHA4 was upregulated in central nervous system neoplasms, breast cancer, head and neck cancer, and melanoma and downregulated in bladder cancer and ovarian cancer (Figure 1(a)). PLEKHA4 was significantly upregulated in glioblastoma (Sun brain and Lee brain) compared to the corresponding normal tissue samples (Figures 1(b) and 1(c)). GEPIA database analysis showed that PLEKHA4 transcript expression in the LGG and GBM tissues was significantly higher compared to the normal brain tissues (Figures 1(d) and 1(e)). Moreover, HPA database analysis indicated that PLEKHA4 protein staining which was positive in the highly malignant glioma cells was higher compared to the normal neuropil area (Figures 2(a) and 2(b)). Consistent with this finding, increased expression of PLEKHA4 was observed in 90% (36/40) of glioma tissues compared with that normal brain tissue samples by IHC (Figures 2(c)–2(f)). The high PLEKHA4 protein level was significantly correlated with age,WHO grade and Ki67, IDH satus, and tumor size (*P* < 0.05; Table S1).

### 3.2. High Expression of PLEKHA4 Predicts Poor Prognosis of Glioma

**Figure 3:**
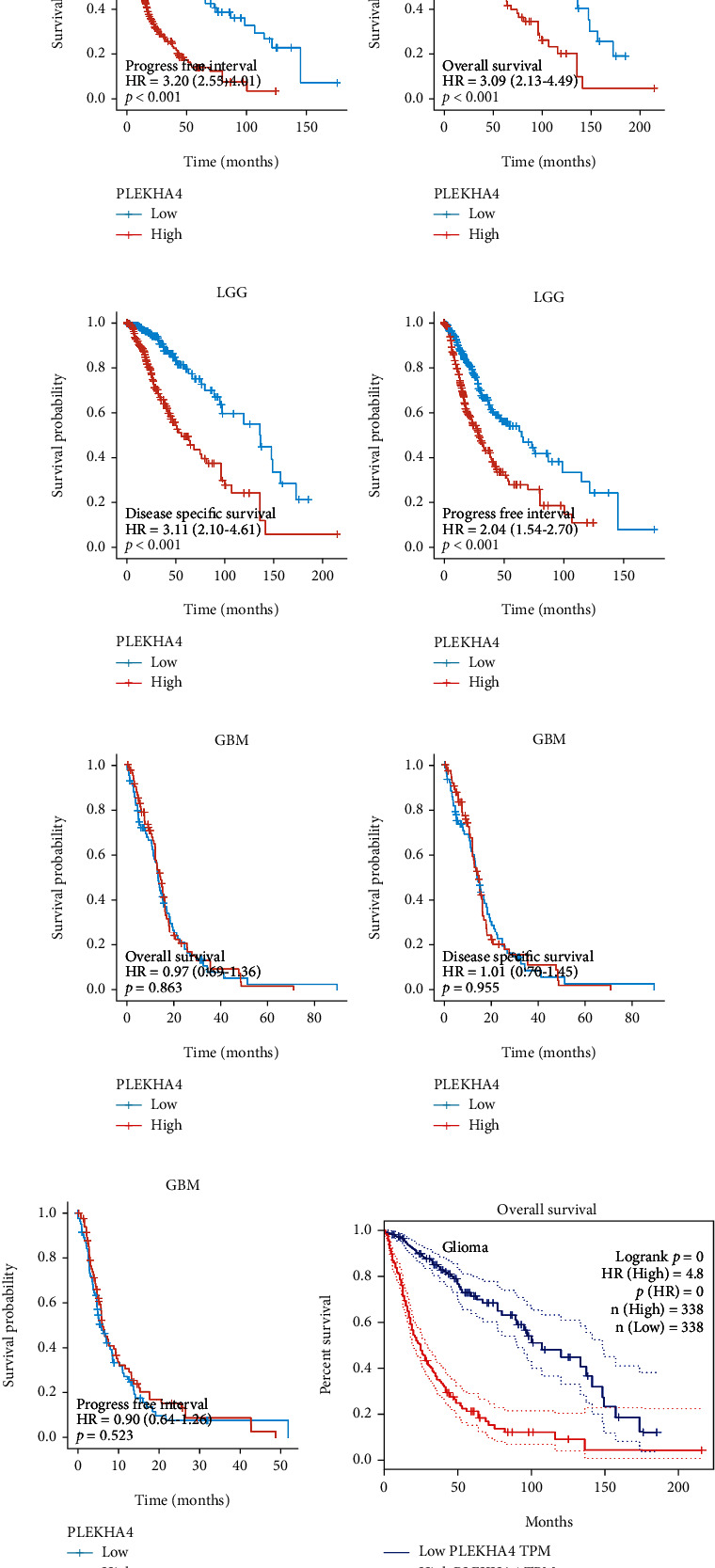
The survival curves of percent survival in different datasets based on the expression level of PLEKHA4. Patients with high PLEKHA4 expression had significantly shorter OS, DSS, and PFI than those with low PLEKHA4 expression in all gliomas (a–c) (703 cases) and LGG (d–f) (529 cases) (*P* < 0.001) in TCGA. But TCGA database had no statistical significance in GBM (g–i) (174 cases) (*P* > 0.05). (j–l) Survival curve of differential PLEKHA4 expression were analyzed in validation set from GEPIA database.

**Figure 4:**
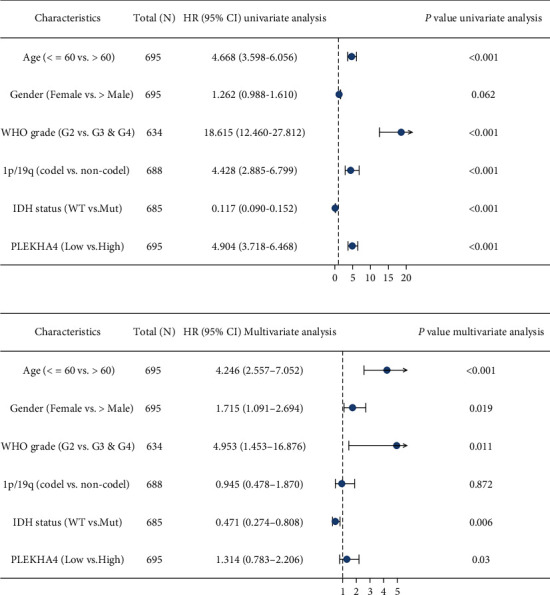
Forest plot for cox proportional hazards model of overall survival (OS) in glioma. (a) Univariate analysis of OS showed that the OS of glioma patients was linked with age, WHO Grade, 1p19q codeletion status, IDH mutation status, and PLEKHA4 expression. (b) Multivariate analysis of OS showed that the OS of glioma patients was linked with age, gender, WHO Grade, IDH mutation status, and PLEKHA4 expression.

Of the 703 cases, removal control/normal (not all items have control/normal) and retention of clinical information, patients with high PLEKHA4 expression had significantly shorter OS, DSS, and PFI than those with low PLEKHA4 expression in all gliomas (Figures 3(a)–3(c), both log-rank *P* < 0.001) and LGG (Figures 3(d)–3(f), both log-rank *P* < 0.001). But TCGA database had no statistical significance in GBM (Figures 3(g)–3(i), both log-rank *P* > 0.05). Likewise, we performed the online website of Gene Expression Profiling Interactive Analysis (http://gepia.cancer-pku.cn/index.html). The result showed that high expression of PLEKHA4 predicted shorter over survival than PLEKHA4 low expression of glioma (Figures 3(j)–3(k), *P* < 0.001). In addition, univariate analysis showed that age (HR 4.668; 95% CI 3.598, 6.056; *P* < 0.001), WHO grade (HR 18.615; 95% CI 12.460, 27.812; *P* < 0.001), 1p/19q codel status (HR 4.428; 95% CI 2.885, 6.799; *P* < 0.001), IDH mutation status (HR 0.117; 95% CI 0.090, 0.152; *P* < 0.001), and PLEKHA4 (HR 4.904; 95% CI 3.718, 6.468; *P* < 0.001) were associated with OS (Figure 4(a)). In multivariate analysis, age (HR 4.246; 95% CI 2.557, 7.052; *P* < 0.001), gender (HR 1.715; 95% CI 1.091, 2.694; *P* = 0.019), WHO grade (for OS, HR 4.953; 95% CI 1.453, 16.876; *P* = 0.011), IDH mutation status (for OS, HR 0.471; 95% CI 0.274, 0.808; *P* = 0.006), and PLEKHA4 (HR 1.314; 95% CI 0.783, 2.206; *P* = 0.030) were associated with OS (Figure 4(b)).

### 3.3. Association between PLEKHA4 Expression and Clinicopathological Features of Patients with Glioma

**Figure 5:**
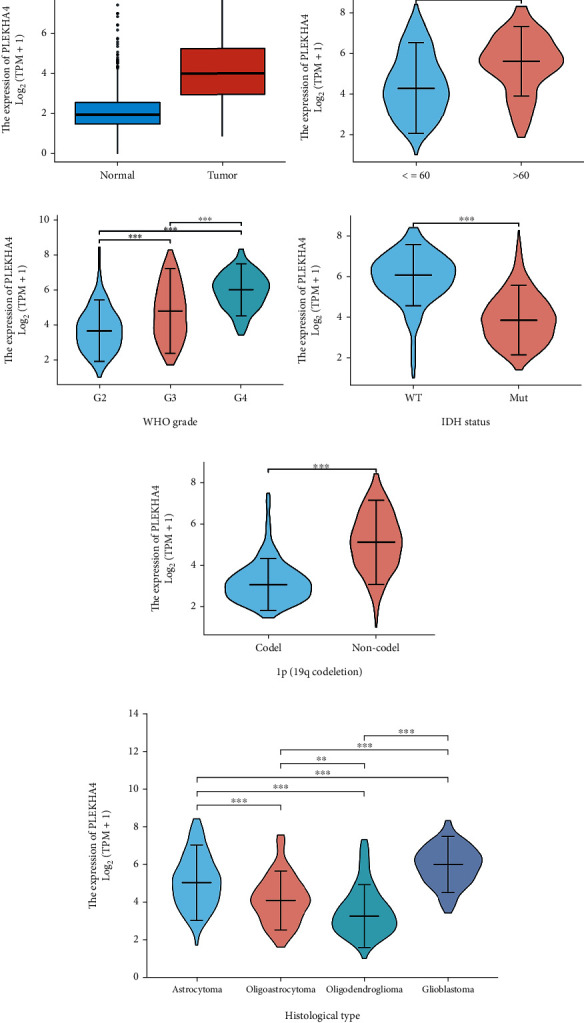
The correlation between PLEKHA4 expression and the clinical characteristics of glioma patients. (a) PLEKHA4 expression levels in glioma (red) and corresponding normal brain tissues (blue) from TCGA datasets. (b–f) Correlation of PLEKHA4 expression with age (b), WHO grade (c), IDH mutation status (d), 1p19q codeletion status (e), and histology type (f).

We downloaded the clinical data of glioma patients from the TCGA database (Table 1) and then used the Wilcoxon rank sum test to analyze the association between PLEKHA4 expression level and clinicopathological variables. PLEKHA4 expression was associated with age (*P* < 0.001), WHO grade (*P* < 0.001), IDH mutation status (*P* < 0.001), 1p19q codeletion status (*P* < 0.001), and histological type (*P* < 0.001) in glioma patients. However, no association was observed between PLEKHA4 expression sex (*P* = 0.491) (Table 1). As shown in Figure 5, expression of PLEKHA4 was significantly increased in glioma tissues compared with normal tissues, and high expression of PLEKHA4 was significantly associated with age over 60 (*P* < 0.001), WHO grade IV (*P* < 0.001), IDH wild type (*P* < 0.001), 1p19q noncodeletion status (*P* < 0.001), and glioblastoma (*P* < 0.001).

### 3.4. PLEKHA4 Expression Is Correlated with Immune Infiltration Levels in Glioma

**Figure 6:**
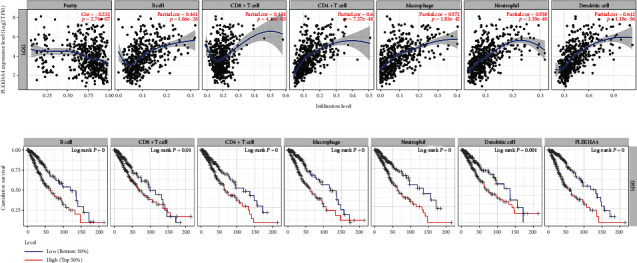
The PLEKHA4 expression level has significant positive correlations with infiltrating levels of B cell, CD4+ T cells, Macrophages, Neutrophils, and DCs in glioma. (a) Positive correlation exists between the PLEKHA4 expression level and infiltrating levels of B cell (*r* = 0.416, *P* = 1.66*e* − 26), CD8+ T cells (*r* = 0.131, *P* = 4.11*e* − 3), CD4+ T cells (*r* = 0.6, *P* = 7.37*e* − 48), Macrophages (*r* = 0.572, *P* = 1.83*e* − 42), Neutrophils (*r* = 0.558, *P* = 3.39*e* − 40), and DCs (*r* = 0.642, *P* = 1.18*e* − 56) in glioma). (b) Cumulative survival is related to B cell, T cells, Macrophages, Neutrophils, and DCs in glioma. (The B cell, T cells, Macrophages, Neutrophils, and DCs are factors related to the cumulative survival rate of glioma over time).

Infiltrating immune cells are important components of the tumor microenvironment and are frequently associated with tumor behavior and patient outcomes [37]. So we assessed the correlation between the expression of PLEKHA4 and the infiltration level of 6 immune cells in glioma through TIMER. The results showed that the expression of PLEKHA4 was positively correlated with the infiltration level of B cells, CD8+ cells, CD4+ T cells, macrophages, dendritic cells, and neutrophils in glioma (Figure 6(a)). In addition, we investigated the prognostic value of different immune cells via TIMER. The Kaplan-Meier survival curves were generated with a 50% split infiltration percentage, and the samples were divided into high-level and low-level groups. The results showed that the levels of infiltrating B cells, CD8+ cells, CD4+ T cells, macrophages, dendritic cells, and neutrophils were related to the cumulative survival rate of glioma patients (Figure 6(b)).

### 3.5. Relationship between PLEKHA4 Expression and Tumor Infiltrating Immune Cells

**Figure 7:**
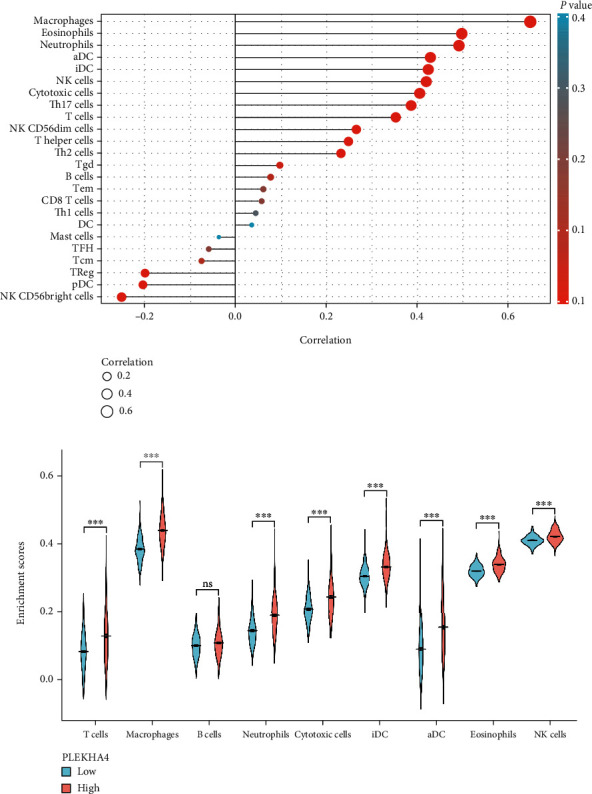
The expression of PLEKHA4 was associated with immune infiltration in the glioma microenvironment. (a) The Lollipop polts showed a positive correlation between PLEKHA4 and 18 immune cells and a negative correlation between PLEKHA4 and 6 immune cell subsets. (b) The violin polts described that T cells, Macrophages, Neutrophils, Cytotoxic cells, iDC, aDC, Eosinophils, and NK cells were apparently increased in high expression group compared with low expression group (ns, *P* ≥ 0.05; ^∗^*P* < 0.05; ^∗∗^*P* < 0.01; ^∗∗∗^*P* < 0.001).

GSVA (Gene Set Variation Analysis), including the ssGSEA immune infiltration algorithm, is a calculation method for estimating the abundance ratio of tumor infiltrating white blood cells in a sample based on gene expression data [38]. We ran ssGSEA within R software. Twenty-four immune cell markers gained from one published article [39]. We analyzed the gene expression data of glioma samples to determine the abundance ratio of 24 types of immune cells. Finally, 703 samples were selected with *P* value < 0.05 and then divided into 2 groups according to the median expression of PLEKHA4. The Spearman was continuously used to evaluate the different concentrations of immune cells in the PLEKHA4-high and PLEKHA4-low expression. As shown in Figure 7, T cells (*P* < 0.001), macrophages (*P* < 0.001), neutrophils (*P* < 0.001), cytotoxic cells (*P* < 0.001), iDC (*P* < 0.001), aDC (*P* < 0.001), eosinophils, (*P* < 0.001) and NK cells (*P* < 0.001) were the main immune cells affected by PLEKHA4 expression. Among them, macrophages (*P* < 0.001) were apparently increased, but NK CD56+ cells (*P* < 0.001) were decreased in the PLEKHA4-high group compared with the PLEKHA4-low group.

### 3.6. Correlation between PLEKHA4 Expression and Immune Cell Biomarkers in Glioma

To further evaluate the role of PLEKHA4 in tumor immunity, we determined the correlation of PLEKHA4 expression with immune cell biomarkers in glioma by GEPIA database. The results showed that PLEKHA4 expression was related with CD4+ T cell biomarker (CD4), CD8+ Tcell biomarkers (CD8A and CD8B), B-cell biomarker (CD19), Neutrophil biomarkers (ITGAM and CCR7), Dendritic cell biomarkers (HLA-DPB1,CD1C and NRP1), NK cell biomarkers (KIR2DL1, KIR2DL4 and KIR3DL3), M1 macrophage biomarkers (NOS2, IRF5 and PTGS2), M2 macrophage biomarkers (CD163,VSIG4 and MS4A4A), mast cell biomarkers (TPSB2 and HDC), Th1 cell biomarker (T-bet), Th2 cell biomarkers (GATA3, STAT6 and STAT5A), and Th17 cell biomarker (STAT3). These findings partially suggested that PLEKHA4 expression had a significant correlation with immune cell infiltration (Table 2).

### 3.7. Relationship between PLEKHA4 and Immune Checkpoints

**Figure 8:**
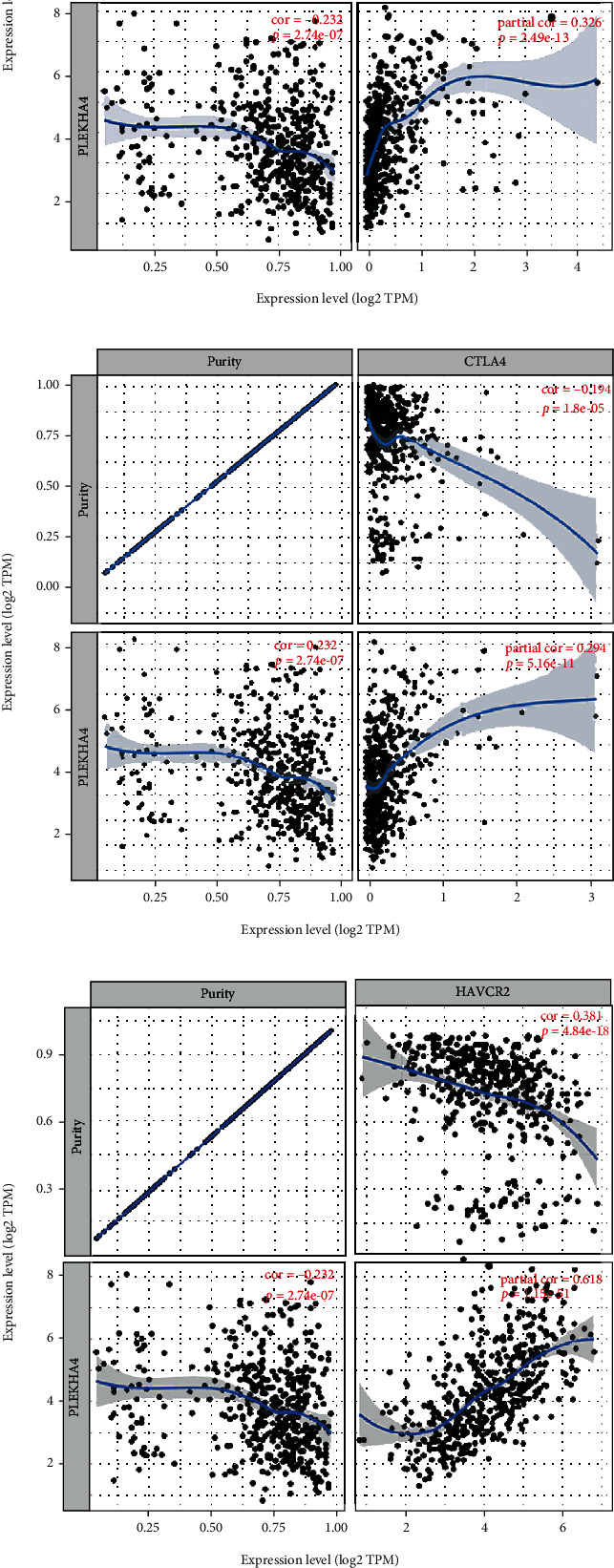
The correlation of PLEKHA4 expression with PD-1, PD-L1, CTLA-4, and TIM-3 expression in glioma. (a) Spearman's correlation of PLEKHA4 with expression of PD-1 in glioma adjusted by purity using TIMER. (b) Spearman's correlation of PLEKHA4 with expression of PD-L1 in glioma adjusted by purity using TIMER. (c) Spearman's correlation of PLEKHA4 with expression of CTLA-4 in glioma adjusted by purity using TIMER. (d) The expression correlation of PLEKHA4 with TIM-3 in glioma adjusted by purity using TIMER. (e) The expression correlation of PLEKHA4 with PD-1 in glioma determined by GEPIA database. (f) The expression correlation of PLEKHA4 with PD-L1 in glioma determined by GEPIA database. (g) The expression correlation of PLEKHA4 with CTLA-4 in glioma determined by GEPIA database. (h) The expression correlation of PLEKHA4 with TIM-1 in glioma determined by GEPIA database (ns, *P* ≥ 0.05; ^∗^*P* < 0.05; ^∗∗^*P* < 0.01; ^∗∗∗^*P* < 0.001).

PD1 (PDCD1), PD-L1 (CD274), CTLA-4, and TIM-3 (HAVCR2) are important immune checkpoints responsible for tumor immune escape. Given that the potential carcinogenic effects of PLEKHA4 in glioma, the relationships between glioma and PD1, PD-L1, CTLA-4, and TIM-3 were analyzed. As shown in Figures 8(a)–8(d), PLEKHA4 expression had a significantly positive correlation with PD1/PDL1, CTLA-4, and TIM-3 in glioma adjusted by purity using TIMER. From the expression correlation analysis, we also found that PLEKHA4 was significantly and positively correlated with PD1/PDL1, CTLA-4, and TIM-3 in glioma (Figures 8(e)–8(h)). These results indicated that tumor immune escape might involve the PLEKHA4-related carcinogenesis of glioma.

### 3.8. PLEKHA4-Related Signaling Pathway Performed on GSEA

**Figure 9:**
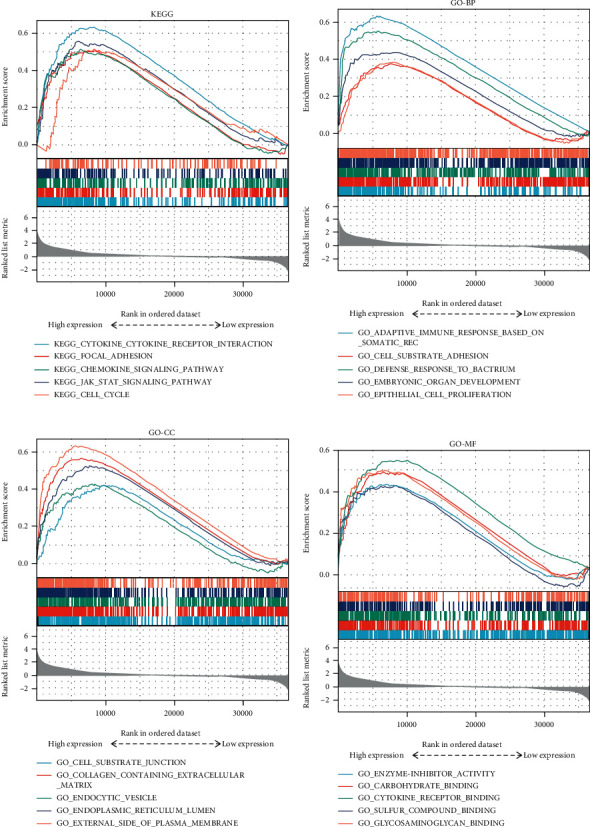
SEA analysis of PLEKHA4. (a) KEGG showed the five most highly enriched signaling pathways in PLEKHA4 high expression. (b–d) GSEA analysis revealed differential enrichment of genes in GO with PLEKHA4 high expression groups. The low expression groups were not showed. (NES = normalized enrichment score, NOM *P* value < 0.05, FDR *q* value < 0.05). Abbreviations: GSEA: Gene Set Enrichment Analysis; GO: Gene Ontology; KEGG: Kyoto Encyclopedia of Genes and Genomes.

Gene Set Enrichment Analysis was performed to explore glioma involved signaling pathways between low and high PLEKHA4 expression groups. The most significantly enriched Kyoto Encyclopedia of Genes and Genomes (KEGG) and Gene Ontology (GO) items were selected according to NES. Several items of KEGG and GO significantly enriched in the high and low PLEKHA4 expression groups, due to the limited space, were shown in Figure 9. Detailed analysis results were listed in additional Table S2. Several biological process items associated with KEGG cytokine receptor interaction, KEGG focal adhesion, KEGG chemokine signaling pathway, KEGG JAK STAT signal pathway, and KEGG cell cycle were enriched in the high PLEKHA4 expression group based on the NES, normal *P* value, and false discovery rate value (Figure 9). The KEGG and GO items associated with nervous system function in the low PLEKHA4 expression group were not in the table.

## 4. Discussion

PLEKHA4, also known as PEPP1, encodes a protein containing the pleckstrin homology (PH) domain located near the N-terminus and contains the putative phosphatidylinositol 3, 4, 5-triphosphate binding motif (PPBM) [40]. Recently, Shami Shah et al. reported that PLEKHA4/kramer attenuates dishevelled ubiquitination to modulate Wnt and planar cell polarity signaling [22]. Elevated expression of this gene has been observed in some melanomas [25]. As far as we know, there is no more literature that described the potential prognostic impact of PLEKHA4 in other tumor, including glioma. As a result, we performed and studied the potential value of PLEKHA4 in glioma, which was the first to analyze the expression of PLEKHA4 in a large number of human glioma patients. In the context of clinical and RNA-seq data, we performed a retrospective analysis of histologically confirmed 703 glioma patients. Our results highlighted that PLEKHA4 mRNA expression was associated with age, IDH mutation status, 1p19q codeletion status, histological type, and the tumor grade in glioma patients.

Here, we detected that PLEKHA4 protein was localized to the cytoplasm, and the expression of PLEKHA4 in glioma tissues was significantly higher than that in adjacent normal tissues by immunohistochemical staining and HPA online tool. Meanwhile, we found that high PLEKHA4 expression in glioma patients from the TCGA database was significantly related to worse histological grade and shorter OS, DSS, and PFI. In this study, we found the correlation of PLEKHA4 expression with survival of glioma patients using GEPIA, an online database. The increased expression of PLEKHA4 was associated with the poor prognosis. We downloaded datasets from TCGA and carried out analysis by R-3.6.3 for the purpose of further investigating the underlying mechanisms and relationships of PLEKHA4 expression in gliomas. As a result, univariate analysis revealed that PLEKHA4 expression related to age, tumor WHO grades, IDH mutation status, 1p19q codeletion status, and histological type. Similarly, multivariate analysis showed that high PLEKHA4 expression positively correlated with age over 60, tumor WHO grades IV, IDH wild type status, and high-grade glioblastoma (GBM). Upregulated expression of PLEKHA4 was an independent prognostic factor for adverse prognosis. Furthermore, we used TCGA data to carry out GSEA. The results showed that cytokine receptor interaction, focal adhesion, chemokine signaling pathway, JAK STAT signal pathway, and cell cycle in KEGG and adaptive immune response based on somatic recombination of immune receptors built from immunoglobulin superfamily domains, cell substrate adhesion, cell substrate junction, and cytokine receptor binding in GO were differentially enriched in PLEKHA4 high expression phenotype. These all suggested that PLEKHA4 may serve as a potential prognostic marker of prognosis and therapeutic target in glioma.

KEGG and GO analyses displayed that PLEKHA4 was mainly associated with cytokines, chemokine and immune response. The development and progression of cancer coincide with changes in the surrounding matrix. Cancer cells can shape their microenvironment functionally by secreting various cytokines, chemokines, and other factors [41]. Activation of JAK-STAT signaling upregulated the levels of tumor-promoting chemokines and cytokines and increased numbers of infiltrating myeloid-derived suppressor cells, thereby promoting tumor growth [42]. There is increasing evidence that innate immune cells (macrophages, neutrophils, dendritic cells, innate lymphoid cells, bone marrow-derived suppressor cells, and natural killer cells) and adaptive immune cells (T cells and B cells) contribute to tumor progression when present in the tumor microenvironment (TME). Tumor infiltrating lymphocytes (TILs) act as important roles in the prognosis and treatment of patients with glioma [43]. TILs can promote or regulate tumor progression and growth through the interaction of different types of cells [44]. The ssGSEA analysis indicated a substantial positive connection of PLEKHA4 expression with infiltration levels of macrophages, neutrophils, T cells, NK cells, and so on in glioma. In this study, we also assessed the immune infiltration based on the expression level of PLEKHA4 and discovered that most immune cells are positively connected with the expression of PLEKHA4 in glioma. Consistently, we carried out the analysis of the correlation with the PLEKHA4 expression level by downloading the data sets of 6 immune-infiltrating cells from the TIMER database. The results showed that PLEKHA4 expression was positively correlated with B cell, CD4+ T cell, CD8+ Tcell, macrophage cell, neutrophil cell, and dendritic cell in glioma. These observations suggested that the glioma microenvironment was immunoactivated, confirming the importance of the immune system in glioma development. The relationships between the expression of PLEKHA4 and different immune cells or immune cell biomarkers implicated that PLEKHA4 might play an important role in regulating tumor immune microenvironment of glioma. Importantly, immune checkpoint inhibitors are effective in many types of solid tumors. Tumor immune checkpoint blockade immunotherapy targeting PD-1/PD-L1 or CTLA-4 prolongs the overall survival of cancer patients [45]. Therefore, we also evaluated the relationship between PLEKHA4 and immune checkpoints. The results showed that the upregulation of PLEKHA4 expression was significantly related to PD-1/PD-L1, CTLA4, and TIM-3 in glioma, indicating that targeting PLEKHA4 may improve immunotherapy in glioma.

In summary, PLEKHA4 might serve as a favourable prognostic factor for patients with glioma. Also, possible key pathways in glioma that were regulated by PLEKHA4 were the chemokine signaling pathway, immune response, JAK STAT signal pathway, and cell cycle. Importantly, our nomogram showed the satisfactory predictive ability for PLEKHA4 alone or in combination with other clinical parameters. In addition, preliminary evidence demonstrated that the immune response was the basis of glioma progression, suggesting a new approach for glioma immunotherapy. Of course, these findings also need basic experiments and further clinical trials to confirm in the future.

## Figures and Tables

**Table 1:** Association between PLEKHA4 expression and clinicopathologic features in the validation cohort.

Characteristic	Low expression of PLEKHA4	High expression of PLEKHA4	*P*
*n*	348	348	
Age, *n* (%)			<0.001
< =60	316 (45.4%)	237 (34.1%)	
> 60	32 (4.6%)	111 (15.9%)	
Gender, *n* (%)			0.491
Female	154 (22.1%)	144 (20.7%)	
Male	194 (27.9%)	204 (29.3%)	
WHO grade, *n* (%)			<0.001
G2	177 (27.9%)	47 (7.4%)	
G3	116 (18.3%)	127 (20%)	
G4	16 (2.5%)	152 (23.9%)	
IDH status, *n* (%)			<0.001
Mut	319 (46.5%)	121 (17.6%)	
WT	26 (3.8%)	220 (32.1%)	
1p/19q codeletion, *n* (%)			<0.001
Codel	159 (23.1%)	12 (1.7%)	
Noncodel	187 (27.1%)	331 (48%)	
Histological type, *n* (%)			<0.001
Astrocytoma	73 (10.5%)	122 (17.5%)	
Oligoastrocytoma	92 (13.2%)	42 (6%)	
Oligodendroglioma	167 (24%)	32 (4.6%)	
Glioblastoma	16 (2.3%)	152 (21.8%)	

**Table 2:** Correlation analysis between PLEKHA4 and biomarkers of immune cells in glioma detected by GEPIA database.

	Biomarker	*R* value	*P* value
CD4+ T cell	CD4	0.32	0
CD8+ T cell	CD8A	0.25	3.4*E* − 11^∗∗∗^
	CD8B	0.19	8.2*E* − 07^∗∗∗^
B cell	CD19	0.16	4.5*E* − 05^∗∗∗^
	CD79A	0.059	0.13
Neutrophil	CEACAM8	0.032	0.41
	ITGAM	0.29	9.5*E* − 15^∗∗∗^
	CCR7	0.15	6.3*E* − 05^∗∗∗^
Dendritic cells	HLA-DPB1	0.40	0^∗∗∗^
	HLA-DQB1	0.33	0^∗∗∗^
	HLA-DRA	0.37	0^∗∗∗^
	HLA-DPA1	0.36	0^∗∗∗^
	CD1C	0.076	0.046^∗^
	NRP1	0.34	0^∗∗∗^
	ITGAX	0.27	1.1*E* − 12^∗∗∗^
NK cells	KIR2DL1	0.091	0.018^∗^
	KIR2DL3	0.079	0.039^∗^
	KIR2DL4	0.26	1.1*E* − 11^∗∗∗^
	KIR3DL1	0.11	0.0028^∗∗^
	KIR3DL2	0.16	3.0*E* − 05^∗∗∗^
	KIR3DL3	-0.0043	0.91
	KIR2DS4	0.049	0.2
M1 macrophage	NOS2	0.16	4.3*E* − 05^∗∗∗^
	IRF5	0.36	0^∗∗∗^
	PTGS2	0.15	5.8*E* − 05^∗∗∗^
M2 macrophage	CD163	0.25	8.4*E* − 11^∗∗∗^
	VSIG4	0.20	2.6*E* − 07^∗∗∗^
	MS4A4A	0.21	6.1*E* − 08^∗∗∗^
Mast cells	TPSB2	0.00049	0.99
	TPSAB1	0.048	0.21
	CPA3	0.037	0.34
	HDC	0.15	0.00011^∗∗∗^
Th1	T-bet	0.3	3.8E − 15^∗∗∗^
	STAT4	-0.13	0.00042^∗∗∗^
	TNF-a	0.02	0.6
Th2	GATA3	0.16	3.3E − 05^∗∗∗^
	STAT6	0.27	3.2E − 13^∗∗∗^
	STAT5A	0.5	0^∗∗∗^
	IL13	0.063	0.099
Th17	STAT3	0.45	0^∗∗∗^
	IL17A	0.061	0.11
Tfh	BCL6	-0.032	0.41

^∗^ *P* value < 0.05; ^∗∗^*P* value < 0.01; ^∗∗∗^*P* value < 0.001.

## Data Availability

The data used to support the findings of this study are included within the article.

## Supplementary Materials

Supplementary 1 Table S1: association between PLEKHA4 expression and clinicopathologic factors in glioma.

Supplementary 2 Table S2: gene sets enriched in phenotype.
