# Supplementary material for: PLEKHA4 Is a Prognostic Biomarker and Correlated with Immune Infiltrates in Glioma
Source: Biomed Res Int. 2023 Jan 17;2023:4504474. doi: 10.1155/2023/4504474 (PMC9881441; doi:10.1155/2023/4504474)
Supplement: Supplementary 1 — Table S1: association between PLEKHA4 expression and clinicopathologic factors in glioma. [file 4504474.f1.docx]

**Table S1** Association between PLEKHA4 expression and clinicopathologic factors in glioma

| Variable | N | PLEKHA4 high expression | PLEKHA4 low expression | p value |
| --- | --- | --- | --- | --- |
| Ages(years) |  |  |  | 0.023 |
| <=60 | 28 | 10 | 18 |  |
| >60 | 12 | 9 | 3 |  |
| Gender |  |  |  | 0.816 |
| female | 14 | 7 | 7 |  |
| male | 26 | 12 | 14 |  |
| Tumor size(mm) |  |  |  | 0.324 |
| <35 | 18 | 7 | 11 |  |
| ≥35 | 22 | 12 | 10 |  |
| WHO grade |  |  |  | 0.024 |
| I-II | 12 | 2 | 10 |  |
| III-IV | 20 | 11 | 9 |  |
| Ki67 positive % |  |  |  | 0.022 |
| ≥15 | 10 | 8 | 2 |  |
| < 15 | 30 | 11 | 19 |  |
