# Supplementary material for: PLEKHA4 Is a Prognostic Biomarker and Correlated with Immune Infiltrates in Glioma
Source: Biomed Res Int. 2023 Jan 17;2023:4504474. doi: 10.1155/2023/4504474 (PMC9881441; doi:10.1155/2023/4504474)
Supplement: Supplementary 2 — Table S2: gene sets enriched in phenotype. [file 4504474.f2.docx]

**Table S2** Gene sets enriched in phenotype

| Gene set name | NES | pvalue | p.adjust | qvalues |
| --- | --- | --- | --- | --- |
| High expression |  |  |  |  |
| KEGG_CYTOKINE_CYTOKINE_RECEPTOR_INTERACTION | 1.985221 | 0.001003 | 0.01393 | 0.010341 |
| KEGG_FOCAL_ADHESION | 1.586321 | 0.001006 | 0.01393 | 0.010341 |
| KEGG_CHEMOKINE_SIGNALING_PATHWAY | 1.592806 | 0.00101 | 0.01393 | 0.010341 |
| KEGG_JAK_STAT_SIGNALING_PATHWAY | 1.716007 | 0.001014 | 0.01393 | 0.010341 |
| KEGG_CELL_CYCLE | 1.572641 | 0.001024 | 0.01393 | 0.010341 |
| GOBP_ADAPTIVE_IMMUNE_RESPONSE_BASED_ON_SOMATIC_RECOMBINATION_OF_IMMUNE_RECEPTORS_BUILT_FROM_IMMUNOGLOBULIN_SUPERFAMILY_DOMAINS | 2.353086 | 0.000999 | 0.017656 | 0.013239 |
| GOBP_CELL_SUBSTRATE_ADHESION | 1.388677 | 0.000999 | 0.017656 | 0.013239 |
| GOBP_DEFENSE_RESPONSE_TO_BACTERIUM | 2.047651 | 0.000999 | 0.017656 | 0.013239 |
| GOBP_EMBRYONIC_ORGAN_DEVELOPMENT | 1.628007 | 0.000999 | 0.017656 | 0.013239 |
| GOBP_EPITHELIAL_CELL_PROLIFERATION | 1.426779 | 0.000999 | 0.017656 | 0.013239 |
| GOCC_CELL_SUBSTRATE_JUNCTION | 1.348195 | 0.000999 | 0.019682 | 0.015141 |
| GOCC_COLLAGEN_CONTAINING_EXTRACELLULAR_MATRIX | 1.82876 | 0.000999 | 0.019682 | 0.015141 |
| GOCC_ENDOCYTIC_VESICLE | 1.364855 | 0.000999 | 0.019682 | 0.015141 |
| GOCC_ENDOPLASMIC_RETICULUM_LUMEN | 1.689614 | 0.000999 | 0.019682 | 0.015141 |
| GOCC_EXTERNAL_SIDE_OF_PLASMA_MEMBRANE | 2.064131 | 0.000999 | 0.019682 | 0.015141 |
| GOMF_ENZYME_INHIBITOR_ACTIVITY | 1.401459 | 0.001 | 0.043354 | 0.035045 |
| GOMF_CARBOHYDRATE_BINDING | 1.604319 | 0.001003 | 0.043354 | 0.035045 |
| GOMF_CYTOKINE_RECEPTOR_BINDING | 1.78929 | 0.001003 | 0.043354 | 0.035045 |
| GOMF_SULFUR_COMPOUND_BINDING | 1.378138 | 0.001004 | 0.043354 | 0.035045 |
| GOMF_GLYCOSAMINOGLYCAN_BINDING | 1.622613 | 0.001005 | 0.043354 | 0.035045 |
